# Environmental factors influencing the diversity and distribution of dictyostelid cellular slime molds in forest and farmland soils of western China

**DOI:** 10.1128/spectrum.01732-23

**Published:** 2023-11-14

**Authors:** Zhaojuan Zhang, Yingkun Yang, Jing Zhao, Yu Li, Steven L. Stephenson, Junzhi Qiu, Pu Liu

**Affiliations:** 1 Engineering Research Center of Edible and Medicinal Fungi, Ministry of Education, Jilin Agricultural University, Changchun, China; 2 Department of Biological Sciences, University of Arkansas, Fayetteville, Arkansas, USA; 3 Key Lab of Biopesticide and Chemical Biology, Ministry of Education, State Key Laboratory of Ecological Pest Control for Fujian and Taiwan Crops, College of Life Sciences, Fujian Agriculture and Forestry University, Fuzhou, Fujian, China; Northwest A&F University, Xianyang, Shannxi, China

**Keywords:** dictyostelids, ecology, environmental factors, soil protists

## Abstract

Dictyostelid cellular slime molds (dictyostelids) are one of the major groups of eukaryotic amoeboid protists occurring in the soil microhabitat. The past few decades have witnessed an unprecedented interest in the ecology of dictyostelids, stimulated in part by the hidden soil protist diversity that has long been overlooked. However, a better understanding of the impact of various environmental factors on the biodiversity of dictyostelids is needed to understand the critical functional role of these organisms in the soil ecosystem. Species diversity of dictyostelids has been surveyed in some provinces of China, but little data exist for Gansu and Guizhou Provinces in western China. In this paper, we report data on the distribution and occurrence of dictyostelids in the soils of these two provinces. Samples were collected from 13 localities in central Gansu and the southeastern and southcentral portions of Guizhou Province. Eleven species of dictyostelids were recovered from 99 samples of soil. Six new records of dictyostelids are reported from Gansu Province and six species from Guizhou Province, respectively. *Polysphondylium violaceum* is a common species in both regions, while *Dictyostelium sphaerocephalum* and *Dictyostelium mucoroides* are widely distributed in Lanzhou, Gansu Province. The total number of species (seven) and the number of endemic species (three) of dictyostelids in mixed forests were higher than those in other forest types. Redundancy analysis indicated that soil pH, temperature, and elevation were the primary factors that affected dictyostelid occurrence. Nonmetric multidimensional scaling and principal component analysis indicated that the dictyostelid communities were significantly different between Gansu and Guizhou Provinces.

**IMPORTANCE:**

Soil protists are an essential yet seriously understudied component of the soil microbiome. In this study, 11 new records of dictyostelids belonging to 2 orders, 3 families, and 4 genera were identified from 99 soil samples collected from different elevations and habitats in central Gansu and the southeastern and southcentral portions of Guizhou Province, China. We found that dictyostelid communities were significantly different between Gansu and Guizhou Provinces, apparently in response to different environmental factors. Moreover, dictyostelids were found to have the highest species diversity in mixed forests. Soil pH, temperature, and elevation were determined to be the primary factors that affect the distribution and occurrence of dictyostelids in Guizhou and Gansu Provinces. This work supplements the survey data available for dictyostelids elsewhere in China. These new findings have significant implications for our understanding of the diversity of soil microorganisms.

## INTRODUCTION

The dictyostelids (phylum Amoebozoa) are a unique soil-dwelling group of transiently multicellular heterotrophic amoebae. Dictyostelids are widely known and studied for their unique lifestyle, in which multicellular fruiting bodies (sorocarps) bearing asexual spores are produced by unicellular amoeboids upon starvation (1). These organisms are ecologically integral to supporting both nutrient recycling and bacterial population control in nature (2, 3). Well-known microhabitats for dictyostelids are the leaf litter decomposition zone of forest soils (4), soils of cultivated regions (5), grasslands (6), deserts (7), and both alpine (8) and arctic tundra (9, 10). Dictyostelids also occur in canopy soil (11) and caves (12). Their community structure and abundance vary among habitats. As such, the community structure of protists, particularly in soils, provides a potential environmental indication of soil health (13).

Compared with other elements of the biota in soil, the classification system used for protozoans and their ecological roles are complex. For the quantitative estimates of soil protozoans such as dictyostelid amoebae, direct observation and culture methods have been used to obtain data (14, 15). In addition, concern over the growing loss of biodiversity makes it possible to use direct surveys of environmental deoxyribonucleic acid (DNA) to obtain real taxonomic data relating to this aspect of microbial ecology (16, 17), which will improve our understanding of microbial diversity in most of the ecosystems on the Earth (18). Therefore, the study of soil protists helps us to develop an in-depth understanding of soil biodiversity resources and to carry out conservation development and utilization to maintain soil and ecosystem health.

Approximately 160 known species of social amoebae have been found to occur in different terrestrial ecosystems. Most species appear to be widespread and can probably be regarded as cosmopolitan in soils, whereas others have a more limited geographical distribution (19). Swanson et al. (20) divided the global distribution of 65 species of forest soil dictyostelids into four categories—cosmopolitan, disjunct, restricted, and pantropical. Although domestic and foreign scholars have conducted resource surveys on dictyostelids in some regions of the world, there are still large areas where no research has been conducted. Thus far, 55 taxa of dictyostelids (54 species and 1 variety) in two orders and four families have been recorded from 25 provinces in China (21). However, there are only three species (*Dictyostelium macrocephalum*, *Dictyostelium implicatum*, and *Dictyostelium crassicaule*) known from Guizhou Province (22), and there are no records from Gansu Province. Knowing the primary factors that impact the diversity and distribution of species is essential for ecological studies of global biodiversity (23, 24). The primary objectives of the present study were to obtain the first information on the species of dictyostelids occurring in some areas of these two provinces and then use data on environmental factors to analyze and compare the distribution and diversity of dictyostelids isolated from these two provinces. The results obtained in the present study appear to provide additional information relating to the importance of environmental factors on the distribution of dictyostelids in nature.

## MATERIALS AND METHODS

### Research area

Lanzhou (35°34′N~37°00′N, 102°36′E~104°35′E) is located in northwest China and central Gansu Province (elevation 1,500–3,000 m and an average annual temperature of 10.3°C, a total area of 131,000 km^2^). It has a temperate continental climate, the average annual sunshine is 2,374 h, the frost-free period is 172 d, and the average annual precipitation is 300 mm (http://www.lanzhou.gov.cn/col/col5/index.html).

Qiandongnan and Qiannan (25°19′N~27°31′N, 107°17′E~109°35′E) are located in southwest China and southeastern Guizhou Province (elevation 800–2,178 m and an average annual temperature of 14°C–18°C℃, a total area of 30,337 km^2^). The autonomous prefecture is within the humid subtropical monsoon climate zone, the average annual sunshine is 1,068–1,296 h, the frost-free period is 270–330 d, and the average annual precipitation is 1,000–1,500 mm (http://www.qdn.gov.cn/zjqdn/zqgk_5935839/zrdl_5871578/; https://www.qiannan.gov.cn/zjqn/zrdl/201812/t20181226_2124264.html).

### Soil sampling and processing

**Fig 1:**
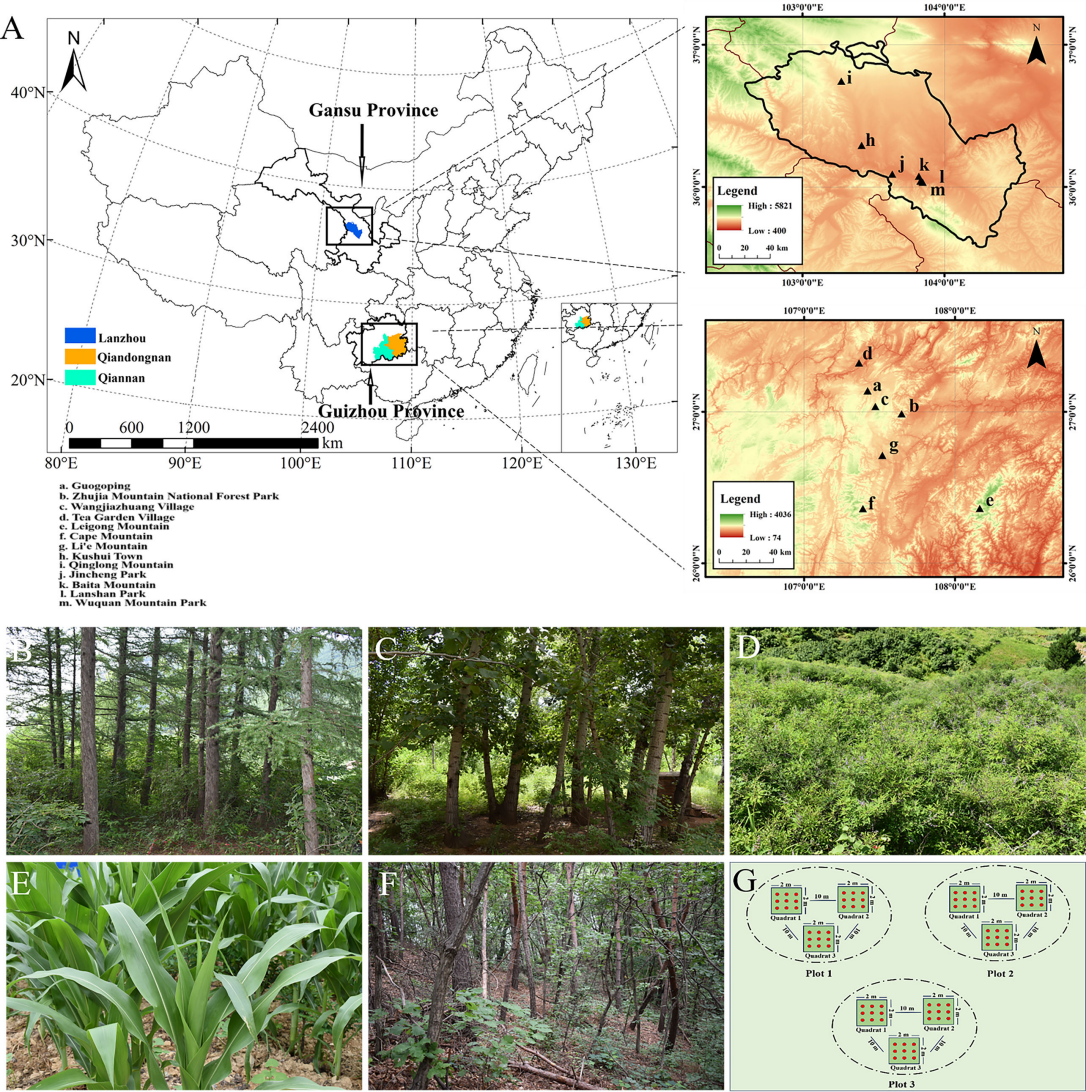
Maps of sampling sites and habitat types. (**A**) Geographical location of sampled localities of six sampling points in Lanzhou City of Gansu Province and seven sampling points in Qiandongnan and Qiannan prefectures of Guizhou Province, China. (**B**) Coniferous forest. (**C**) Broadleaf forest. (**D**) Scrub. (**E**) Farmland. (**F**) Mixed forest. (**G**) Nine-point sampling method. Note: the maps were drawn by ArcGIS software with review number GS(2021)5448, sourced from http://bzdt.ch.mnr.gov.cn/.

(i) From July to August 2020 and February 2021, 13 selected localities of Gansu and Guizhou, China, were sampled (Fig. 1A through F; Table 1). (ii) Sampling (nine-point sampling method, Fig. 1G) involved setting up three sample plots in the same forest type, and three 2 × 2 m quadrats were established for each plot for repetition. The interval between each quadrat was about 10 m, and nine points in the quadrat for sampling were randomly selected, mixed samples from nine sampling points were combined into one sample, and about 100 g of this was placed into disposable sterile ziplock bags, marked with a number, latitude and longitude, elevation, date, vegetation type, temperature, and weather conditions. (iii) For soil preservation, the collected soil was brought back to laboratory quickly, numbered and recorded in the laboratory soil sample database, and then placed in a 4°C℃ refrigerator.

**TABLE 1:** Location information and density for dictyostelid environmental sampling in Gansu and Guizhou Provinces^*a*^

Soil nos.	Localities	No. of soil samples	Coordinates	Elevation (m)	Habitat	Temperature	pH	Density of dictyostelid (clones/g)
			**Guizhou Province**					
6443–6445	Guogoping (a)	3	27°8′15″N, 107°25′15″E	969.18	Scrub	23°C–25°C	4.57	0
6446–6454	Zhujia Mountain National Forest Park (b)	9	26°58′48″N, 107°38′32″E	871.49	Coniferous forest	26°C	5.19	42
6455–6463	Wangjiazhuang Village (c)	9	27°02′03″N, 107°28′27″E	1,247.15	Coniferous forest	22°C–27°C	4.68	42
6464–6472	Tea Garden Village (d)	9	27°19′19″N, 107°21′49″E	741.31	Scrub	21°C–29°C	5.8	25
6473–6481	Leigong Mountain (e)	9	26°22′23″N, 108°10′45″E	1,538.41	Broadleaf forest	19°C–31°C	4.72	33
6586–6588	Cape Mountain (f)	3	26°22′31″N, 107°22′3″E	1,260.39	Mixed forest	10°C–21°C	5.31	67
6589–6591	Li’e Mountain (g)	3	26°42′41″N, 107°30′58″E	1,017.39	Mixed forest	9°C–11°C	5.98	34
			**Gansu Province**					
6518–6526	Kushui Town (h)	9	36°17′34″N, 103°24′59″E	1,647.4	Farmland	—6°C–13°C	8.25	28
6527–6535	Qinglong Mountain (i)	9	36°44′15″N, 103°15′56″E	2,153.4	Broadleaf forest	—3°C–10°C	8.42	122
6536–6544	Jincheng Park (j)	9	36°5′10″N, 103°37′27″E	1,584.3	Mixed forest	—3°C–2°C	7.95	28
6545–6553	Baita Mountain (k)	9	36°4′2″N, 103°48′54″E	1,544.3	Mixed forest	—3°C–2°C	8.22	50
6554–6562	Lanshan Park (l)	9	36°4′3″N, 103°52′56″E	2,133.4	Coniferous forest	—3°C–2°C	8.13	33
6563–6571	Wuquan Mountain Park (m)	9	36°6′3″N, 103°46′56″E	1,638.6	Coniferous forest	—3°C–2°C	8.33	6
Total		99						

^*a*^ The letters in parentheses refer to the order of the collection sites in this study, which are consistent with those in Fig. 1.

Using the method of Cavender and Raper (25), the sample material was suspended in distilled water at a ratio of 1:25. The pH values of the soil dilutions were determined with a PHS-3C pH meter. A 0.5 mL portion of the diluted soil suspension and 0.4 mL of *Escherichia coli* were added to hay agar plates separately, and the plates were cultured at 23°C with a 12 h light and dark cycle under aseptic conditions. Each soil sample was set up in three parallels, each plate contained 0.02 g of soil, and the total soil of the three plates was 0.06 g. After 7 d of culture, counts were made of the number of dictyostelid clones on each plate. According to the formula “the clones of dictyostelids per gram of soil sample = the total number of clones of dictyostelids on three plates / 0.06” (26), the density of dictyostelids in the soil sample was obtained.

### Taxonomy

For morphological observations, in the primary isolation plates, the location of each early aggregating clone and sorocarp(s) that developed was marked. The characteristic stages in the life cycle, including cell aggregation and the formation of pseudoplasmodia and sorocarps, were observed under a Zeiss dissecting microscope (Axio Zoom V16) with a 1.5× objective and 10× ocular. Slides with sorocarps were prepared with water as the mounting medium. Features of spores, sorophores, and sorocarps were observed and measured on the slides using a Zeiss light microscope (Axio Imager A2), with 10× ocular and 10, 40, and 100× (oil) objectives. Photographs were obtained with a Zeiss Axiocam 506 color microscope camera. For strain preservation, a purified dictyostelid sorus was picked up with an inoculation needle and packed into HL5 (27) medium, stored at −80°C in the herbarium of the Mycological Institute of Jilin Agricultural University (HMJAU), Changchun, China.

For DNA isolation, PCR amplification, sequencing, and phylogenetic analysis, the molecular methods and classification system used in the present study followed those described by Liu et al. (28) and Sheikh et al. (29). The spores of all isolates being studied were collected with a sterile tip and mixed with the lysis buffer of the MiniBEST universal genomic DNA extraction kit ver.5.0 (TaKaRa, Japan) according to the manufacturer’s protocol. The genomic DNA solution was used directly for the small-subunit (SSU) PCR amplification using the primers 18S FA (AACCTGGTTGATCCTGCCAG) and 18S RB (TGATCCTTCTGCAGGTTCAC) (30). PCR products were sent to Sangon Biotech Co., Ltd. (Shanghai, China) for sequencing. Sequences obtained were deposited in the GenBank database. The newly generated sequences were checked and then submitted to GenBank. All the sequences of closely related species were downloaded from GenBank for phylogenetic analysis to determine their phylogenetic relationships with other taxa in the group (Table 2).

**TABLE 2:** NCBI GenBank accession information for SSU sequences of all isolates included in the phylogenetic analysis^*a*^

Taxon	Isolate no.	Accession no.	Taxon	Isolate no.	Accession no.
*Dictyostelium multiforme*	PL-2018b	MG490371.1	***P . violaceum***	**Se**	**OP310969**
			*P . patagonicum*		GQ496156.1
***D. multiforme***	**T23**	**OP311638**	*Raperostelium stabile*	ALP-2019a	MN338957.1
*D. minimum*	PL-2018a	MG490369.1			
*D. giganteum*	WS589	AM168042.1	*R. minutum*	Feb-71	AM168051.1
***D. giganteum***	**N51**	**OP310835**	*R. ibericum*	214rjb	HQ141495.1
			*Hagiwaraea coeruleostipes*	CRLC53B	AM168036.1
*D. purpureum*	QSpu36	FJ424828.1			
***D. purpureum***	**Sd**	**OP310944**	*H. vinaceofusca*	CC4	AM168062.1
***D. purpureum***	**LA**	**OP314172**	*H. lavandula*	B15	AM168047.1
			*Tieghemostelium menorah*	M1	AM168073.1
*D. firmibasis*	TNS-C-14	AM168041.1			
***D. firmibasis***	**Y31**	**OP311412**	*T. unicornutum*	MR-2011l	JF892725.1
*D. mucoroides*	sweden 20	HQ141482.1	*T. montium*	MR-2011h	JF892717.1
			*Speleostelium caveatum*	WS695	AM168077.1
***D. mucoroides***	**Q93**	**OP390235**			
***D. mucoroides***	**Q12**	**OP390237**	*Coremiostelium polycephalum*	MY1-1	AM168056.1
***D. mucoroides***	**X31**	**OP390269**	*Heterostelium pallidum*	TNS-C-98	AM168103.1
***D. mucoroides***	**X32**	**OP390285**	***H. pallidum***	**SC**	**OP310943**
***D. mucoroides***	**Q91**	**OP390455**	***H. pallidum***	**Lc1**	**OP311669**
***D. mucoroides***	**L72**	**OP390454**	***H. pallidum***	**WB**	OQ345561
***D. mucoroides***	**N82**	**OP390643**	*H. tenuissimum*	TNS-C-97	AM168105.1
***D. mucoroides***	**T81**	**OP390737**	***H. tenuissimum***	**Ld1**	**OP310821**
*D. sphaerocephalum*	GR11	AM168068.1	***H. tenuissimum***	**Lc2**	**OP311727**
***D. sphaerocephalum***	**Q13**	**OP392881**	*Acytostelium amazonicum*	HN1B1	HQ141511.1
***D. sphaerocephalum***	**Q63**	**OP392892**			
***D. sphaerocephalum***	**L61**	**OP392943**	*A. leptosomum*	212rjb	HQ141512.1
***D. sphaerocephalum***	**n1**	**OP310822**	*A. singulare*	FDIB	HQ141514.1
***D. sphaerocephalum***	**T31**	OP392986	*Rostrostelium ellipticum*	AE2	AM168112.1
***D. sphaerocephalum***	**W21**	**OP392974**			
***D. sphaerocephalum***	**T22**	**OP392991**	*C. delicata*	TNS-C-226	AM168093.1
***D. sphaerocephalum***	**T21**	**OP392994**	***C. delicata***	**D5**	**OP310071**
*D. macrocephalum*	B33	AM168049.1	*Cavenderia aureostipes*	YA6	AM168083.1
***D. macrocephalum***	**Q512**	**OP311593**			
*D. longosporum*	TNS-C-109	AM168048.1			
*Polysphondylium laterosorum*	AE4	AM168046.1	*C. fasciculata*	SmokOW9A	AM168086.1
			*C. parvispora*	OS126	AM168091.1
*P. violaceum*	P6	AM168108.1	*C. exigua*	TNS-C-199	AM168085.1
***P. violaceum***	**L1**	**OP310802**	*Synstelium polycarpum*	VE1b	AM168057.1
***P. violaceum***	**X1**	**OP310985**	*Physarum polycephalum*	CL	X13160.1
***P. violaceum***	**C21**	**OQ348111**			

^*a*^ New sequences from the present study are indicated in bold.

### Data analysis

In order to compare the distribution of dictyostelids with respect to elevation and habitats as a whole, the relative abundance dataset of dictyostelid species was subdivided into five elevation ranges (700–1,000 m, 1,000–1,300 m, 1,300–1,600 m, 1,600–1,900 m, and 1,900–2,200 m) and five habitat types (scrub, coniferous forest, broadleaf forest, mixed forest, and farmland). The relative abundance (RA) (%) of each species was determined by dividing the number of clones for each species of dictyostelid by the total number of all dictyostelid clones. We used Origin 2021 (OriginLab, Northampton, MA, USA) to draw the bidirectional bar chart to describe the relative abundance of dictyostelid species.

**Fig 2:**
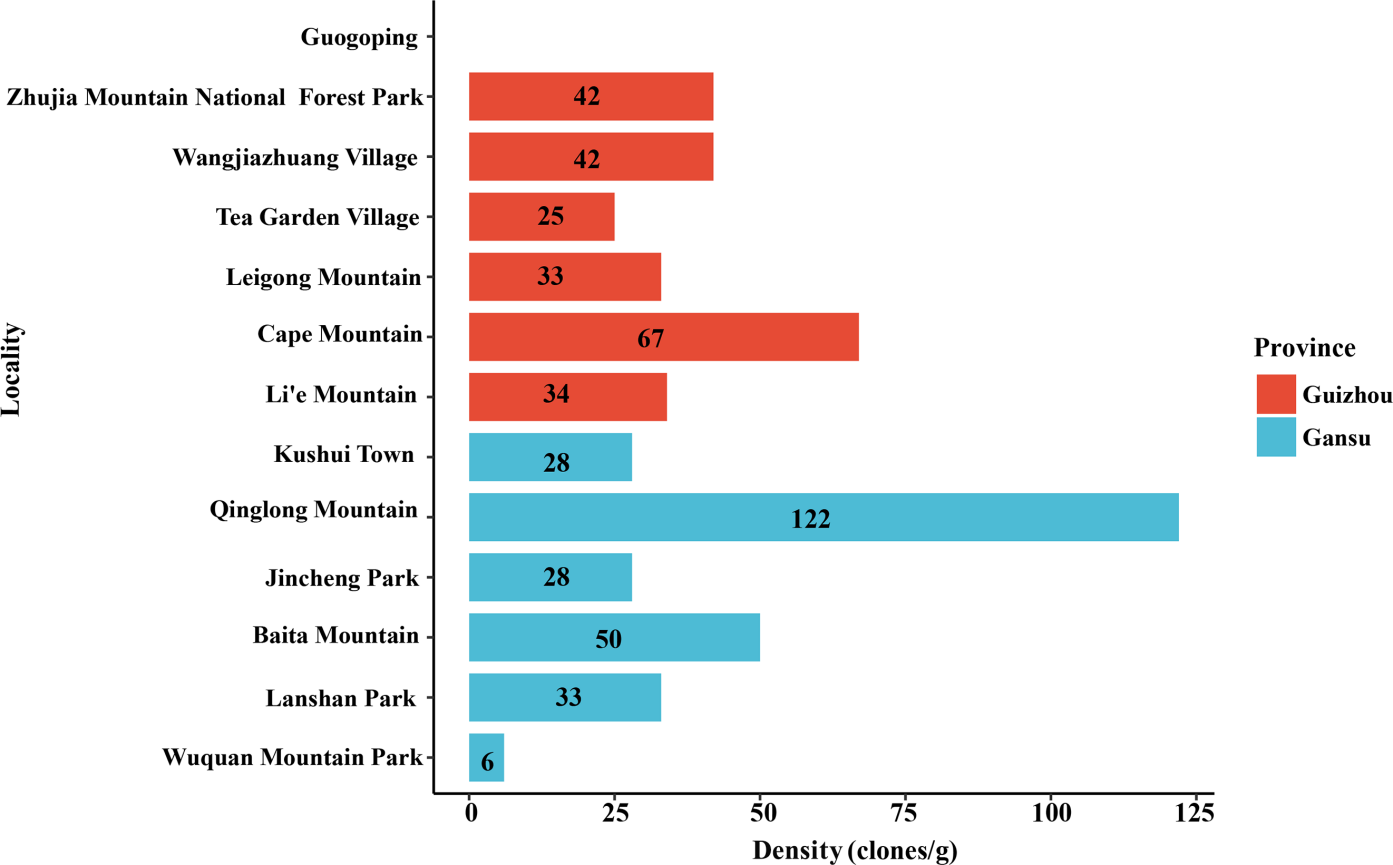
The density of dictyostelids from different collecting sites in Gansu and Guizhou Provinces.

**Fig 3:**
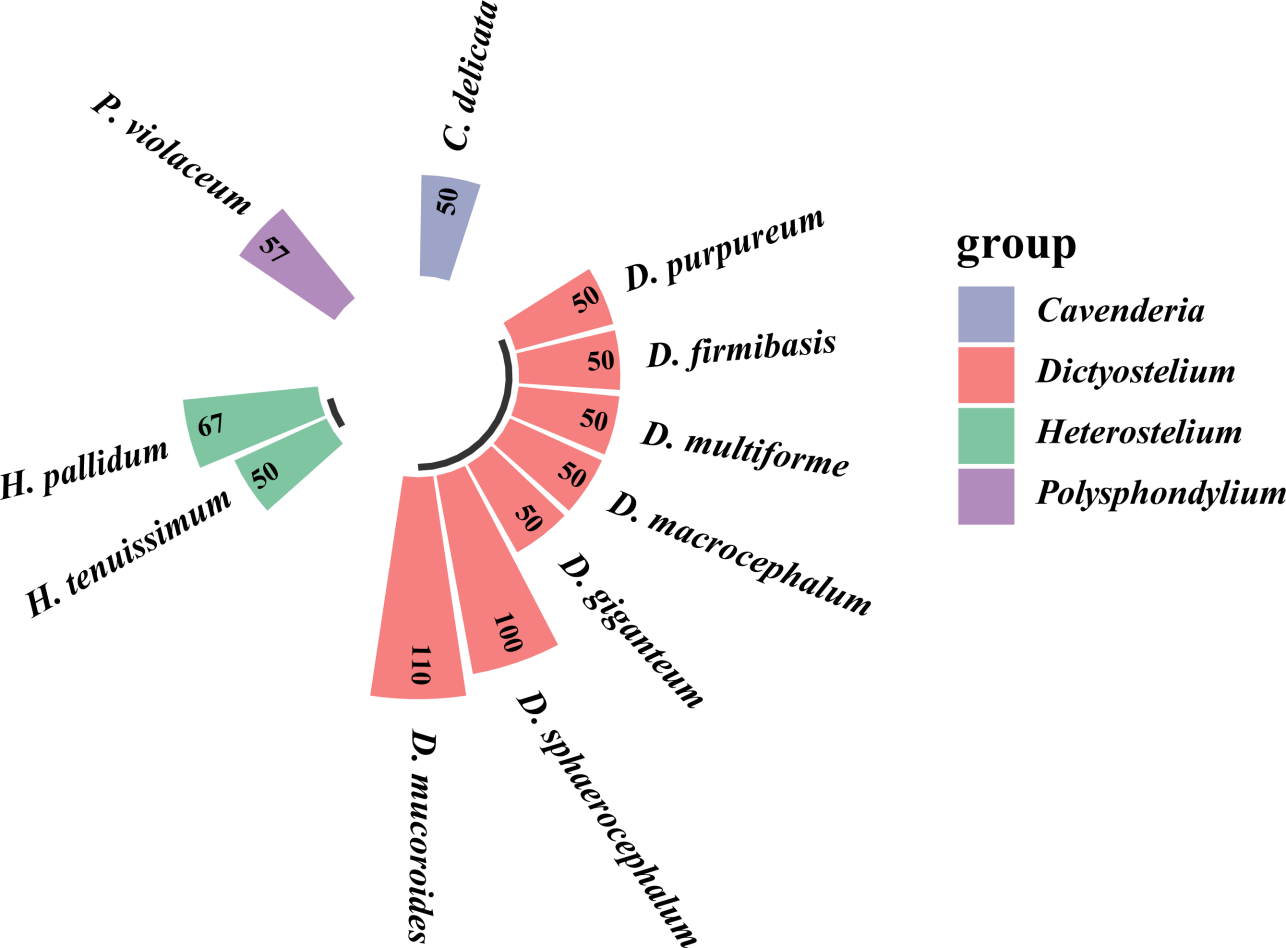
The density (clones/g) of dictyostelid species in different genera from soil samples collected in Gansu and Guizhou Provinces in this study.

**Fig 4:**
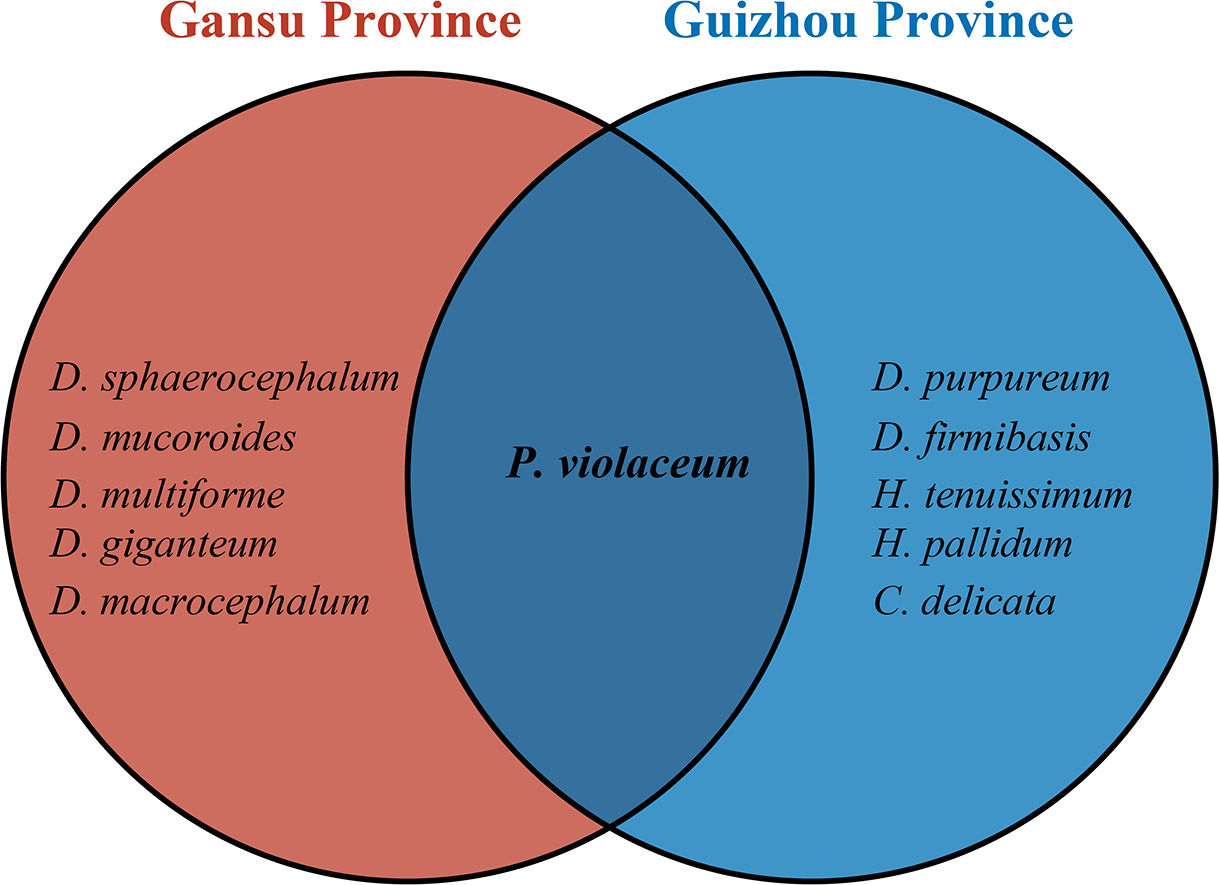
Venn diagram of the distribution of dictyostelids in Gansu and Guizhou Provinces at species levels in this paper.

**Fig 5:**
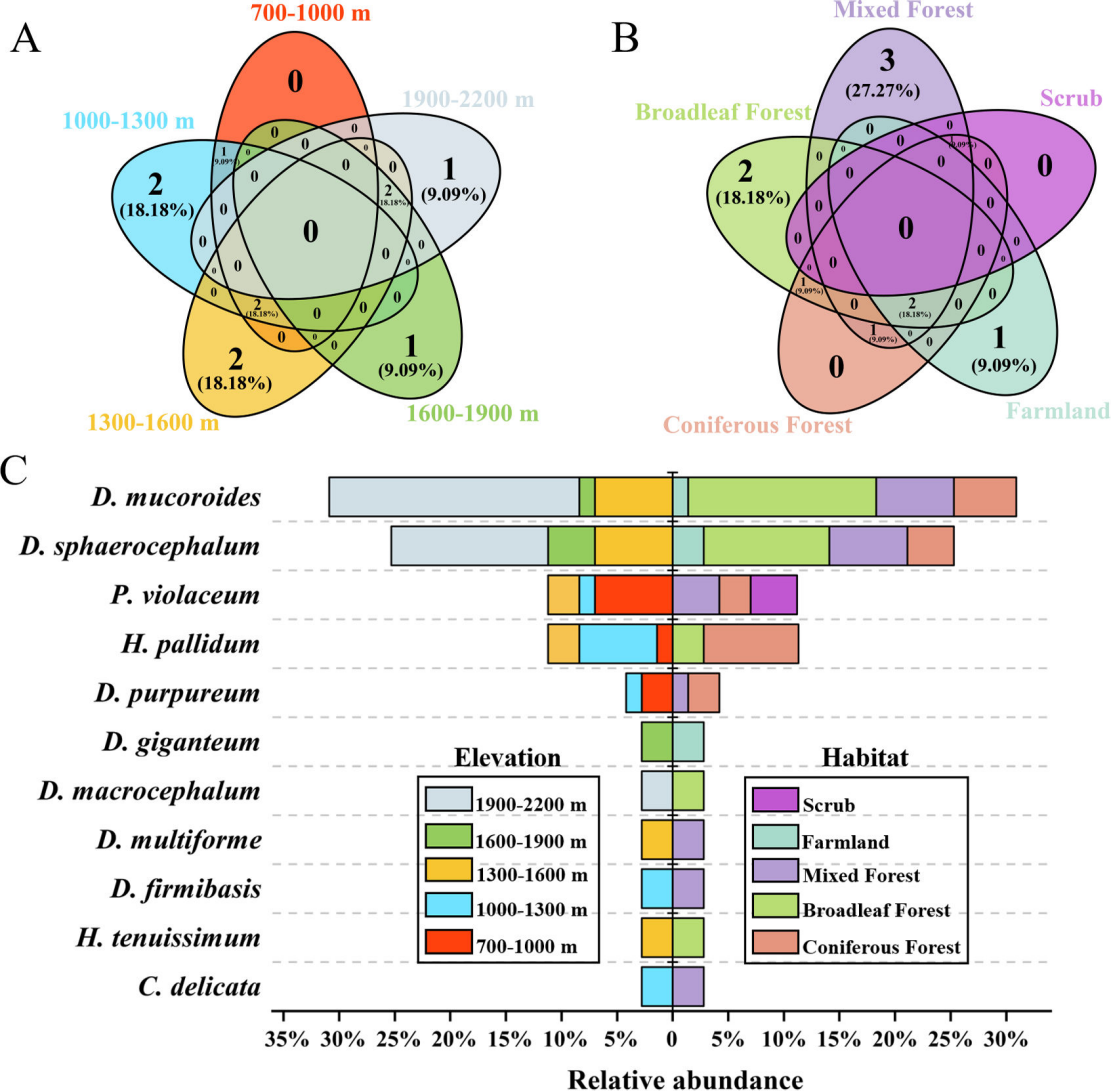
Venn diagrams of the dictyostelid communities of (**A**) five elevation ranges and (**B**) five habitats and (**C**) the bidirectional bar chart of species distribution with relative abundance. Note: the relative abundance (%) of each species was determined by dividing the number of clones for each species of dictyostelid by the total number of all dictyostelid clones.

A histogram (Fig. 2) was generated to evaluate the differences in dictyostelid densities for all the localities, a ring bar chart (Fig. 3) of dictyostelid density in different genera was used to evaluate the dominant species in each genus, and Venn diagrams (Fig. 4 and 5) of dictyostelid species were used to evaluate species distribution using hiplot (available online: https://hiplot. com. cn/). Histogram data and ring bar chart data were derived from the density of dictyostelids. Venn data included the number of species per habitat type and per elevation range and the number of species of dictyostelids in the two provinces.

**Fig 6:**
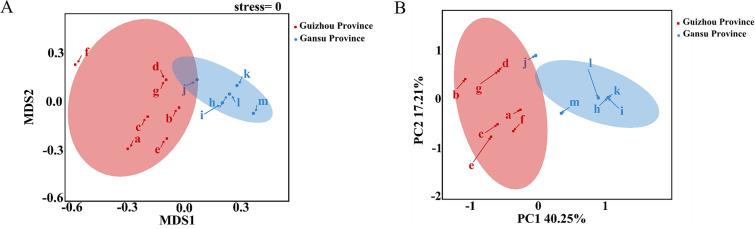
NMDS developed using Bray–Curtis distance (**A**) and PCA (**B**) of different sampling sites in Gansu and Guizhou Provinces. The red circles represent the species of dictyostelids collected from different soil samples in Guizhou, and the blue circles represent the species of dictyostelids collected from different soil samples in Gansu. The letters of acquisition sites in the figure are the same as those in Table 1.

In order to evaluate the structural differences in soil dictyostelid communities, β*-*diversity analysis, the R-packages “vegan” (31) and “ggplot2” were used to perform nonmetric multidimensional scaling (NMDS) (Fig. 6A) based on Bray–Curtis distances and principal component analysis (PCA) (Fig. 6B) to detect the influence of different sites on the dictyostelid community, as well as to make further comparisons between groups.

**Fig 7:**
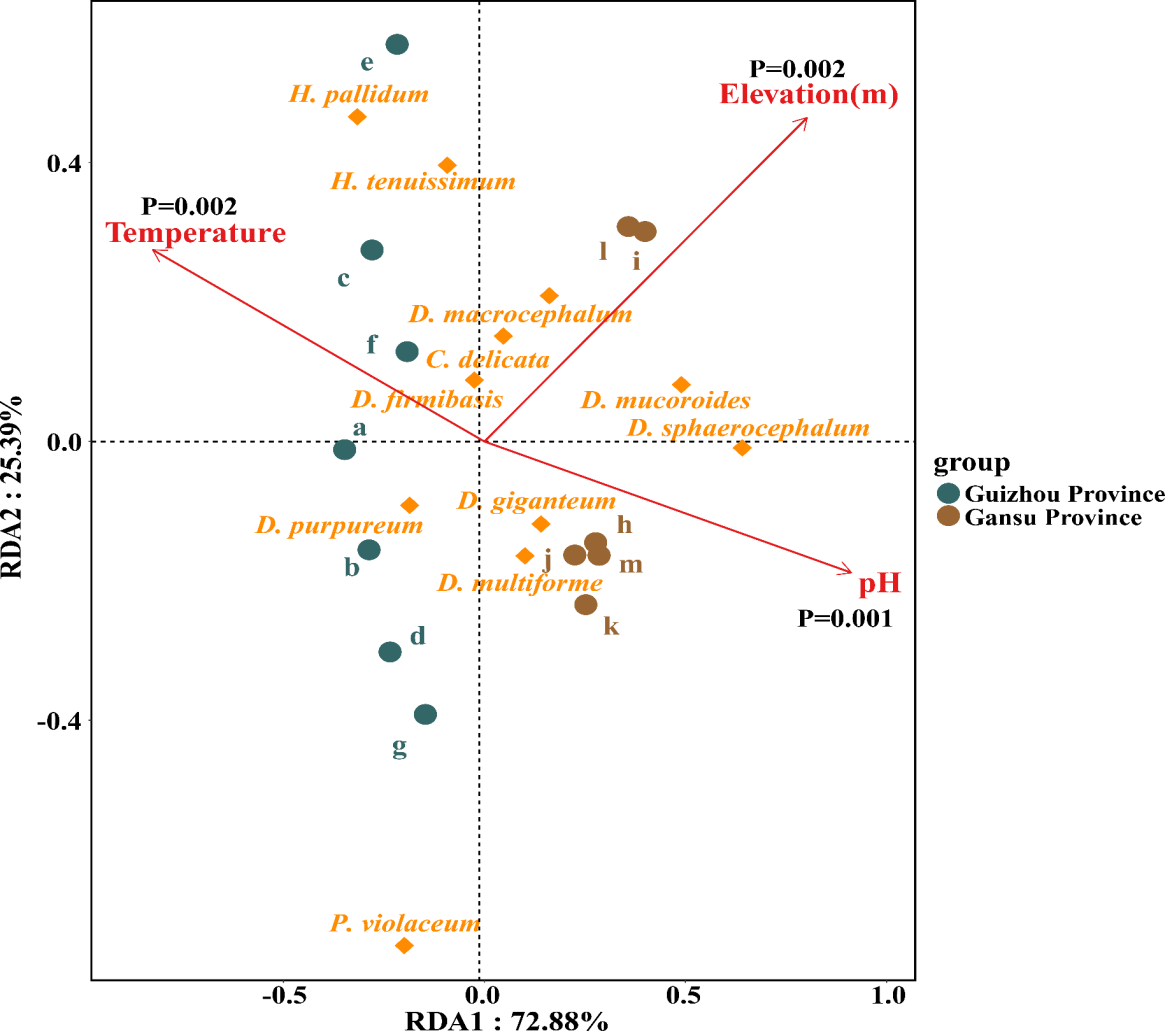
Redundancy analysis of the relationships between the dictyostelid species from 13 sampling sites in Gansu and Guizhou Provinces with environmental factors. Correlations between environmental variables were represented by the length and angle of arrows (the alphabetical order of the collection points in the figure is in accordance with the order in Table 1).

The inﬂuence of environmental factors on the dictyostelid communities of the collecting locality was analyzed by redundancy analysis (RDA) (Fig. 7) using the R-package “vegan.” Environmental factors included temperature, pH, and elevation.

## RESULTS

### Characteristics of species composition

#### Species of dictyostelids from different sampling localities

A total of 71 clones of dictyostelids were recovered from the 99 collected soil samples. Soil samples came from 13 different localities representing five different habitat types, including scrub, coniferous forest, broadleaf forest, mixed forest, and farmland (Fig. 1 and Table 1). The 99 soil samples were not equally productive for dictyostelids. No isolates were recovered from the Guogoping locality in Guizhou Province, but dictyostelids were recorded from the other 12 localities. The density of dictyostelids was different in different localities, with the highest density of dictyostelids recorded for a single collecting site on Qinglong Mountain in Gansu Province (collected from a broadleaf forest at 36°44′15″N, 103°15′56″E) of 122 clones/g at this locality (Fig. 2). The density of dictyostelids from 12 other collecting sites was always less than 70 clones/g, which suggested that the frequency of occurrence of dictyostelids in soil at these localities was not particularly high.

**Fig 8:**
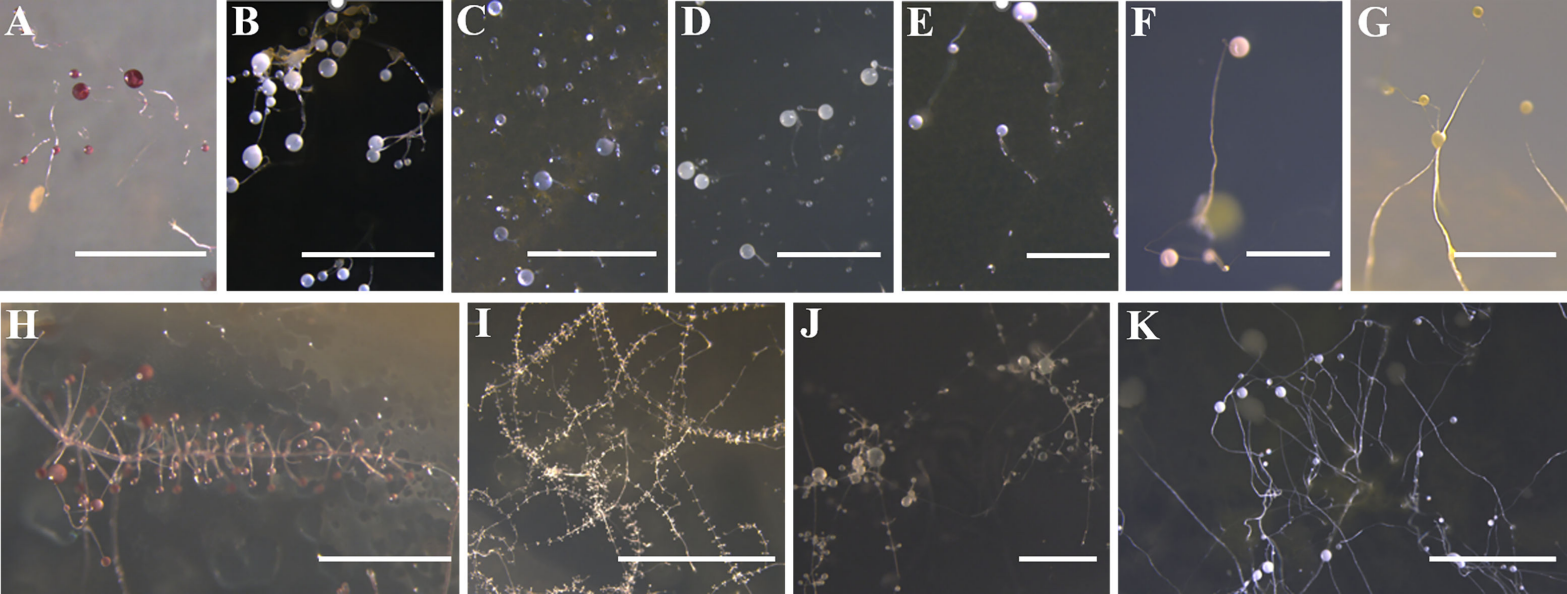
Morphological features of fruiting bodies of 11 species of dictyostelids from Gansu and Guizhou Provinces in this study. *Dictyostelium:* (**A**) *D. purpureum*, (**B**) *D. macrocephalum*, (**C**) *D. sphaerocephalum*, (**D**) *D. mucoroides*, (**E**) *D. multiforme*, (**F**) *D. giganteum*, and (**G**) *D. firmibasis. Polysphondylium:* (**H**) *P. violaceum. Heterostelium:* (**I**) *H. tenuissimum*, (**J**) *H. pallidum. Cavenderia:* (**K**) *C. delicata*. Scale bars: A, B, C, D, K = 2 mm; E, F, G, H, J = 1 mm; I = 5 mm.

**Fig 9:**
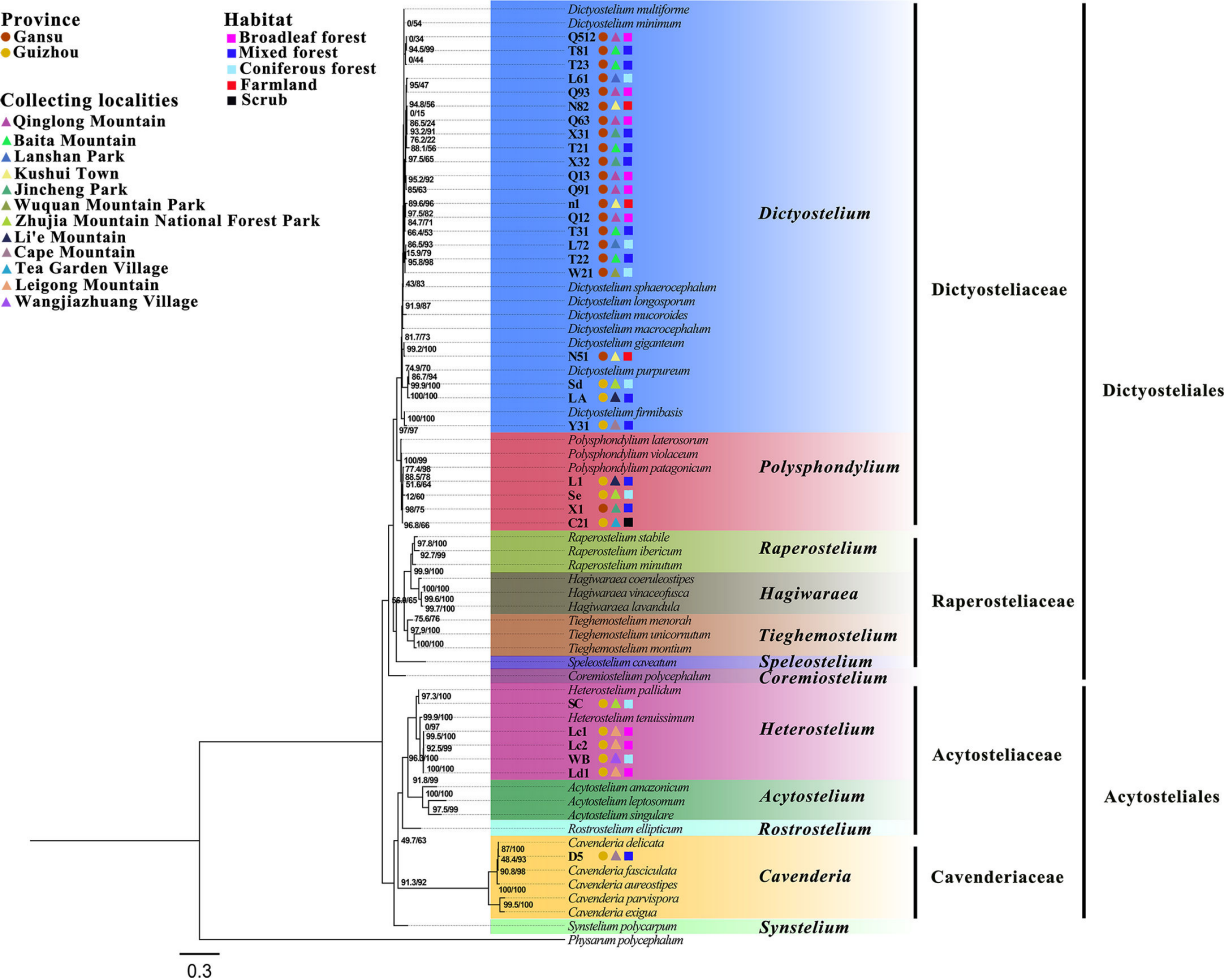
Phylogenetic tree of the 11 species obtained in the present study along with other closely related species of dictyostelids based on SSU rRNA. Numbers in parentheses are SH-aLRT support (%)/ultrafast bootstrap support (%). We used circles to represent dictyostelids from two different provinces, triangles to represent the obtained dictyostelids from different collection sites, and squares to represent dictyostelids from five habitats in this study. Scale bar = 0.3.

In the present study, dictyostelid clones isolated from the samples represented 11 described species; the density (clones/g) for each species is shown in Fig. 3. These species were identified based on both morphology and SSU phylogenetic analyses. Seven species from the genus *Dictyostelium* (sorocarps colorless or with white to pale yellow/purple sori, solitary but sometimes clustered, unbranched sometimes with irregular branches) (Fig. 8A to G), one from *Polysphondylium* (sorocarps with purple sori, with regularly spaced whorls, solitary) (Fig. 8H), two from *Heterostelium* (sorocarps white-hyaline, solitary or tightly clustered, sparsely branched or with whorls of regularly spaced branches) (Fig. 8 I to J), and one from *Cavenderia* (sorocarps white-hyaline, tightly clustered, irregularly branched) were recorded (Fig. 8K). The taxa of this study were identified with the sequence characteristics of the only available marker SSU rRNA for most dictyostelids (i.e., the most widely used taxonomic molecular marker for eukaryotes). The 32 sequences obtained in this study are clustered in the evolutionary branches of the four genera *Dictyostelium*, *Polysphondylium*, *Heterostelium*, and *Cavenderia*, which were consistent with the morphological results (Fig. 9; Table 2). *Dictyostelium sphaerocephalum*, *D. mucoroides*, *D. purpureum*, *D. firmibasis*, *D. multiforme*, *D. macrocephalum*, *D. giganteum*, *Heterostelium tenuissimum*, *H. pallidum*, *Polysphondylium violaceum*, and *Cavenderia delicata* were new records for Gansu and Guizhou Provinces. Six species of dictyostelids were isolated from the two provinces, respectively, and there was only one species in common. Six species of dictyostelids (*D. sphaerocephalum*, *D. mucoroides*, *D. multiforme*, *D. macrocephalum*, *D. giganteum*, and *P. violaceum*) were isolated from Gansu Province, and these were new records for Gansu Province. Six species of dictyostelids (*D. firmibasis*, *D. purpureum*, *H. tenuissimum*, *H. pallidum*, *P. violaceum*, and *C. delicata*) were isolated from Guizhou Province and represented new records for the province (Fig. 4).

#### β*-*Diversity of dictyostelid species

NMDS and PCA of the dictyostelid communities revealed significant differences between soil collection sites in Gansu and Guizhou (stress = 0; Figure 6), based on the dataset used to cluster the samples at the species level. It can be seen that there are significant differences in grouping among sampling points within Guizhou.

### Characteristics of species distribution

#### Relationship of species distribution of dictyostelids with elevation and habitat

The elevation range of this survey is 700–2,200 m, which can be divided into five ranges (700–1,000 m, 1,000–1,300 m, 1,300–1,600 m, 1,600–1,900 m, and 1,900–2,200 m). Two species (*C. delicata* and *D. firmibasis*) occurred only from elevation 1,000–1,300 m; two species (*H. tenuissimum* and *D. multiforme*) occurred only from elevation 1,300–1,600 m, accounting for 18.18%; one species (*D. giganteum*) occurred only from elevation 1,600–1,900 m; and one species (*D. macrocephalum*) occurred only from elevation 1,900–2,200 m, accounting for 9.09%. No species occurred in common at all five elevations (Fig. 5A).

In this study, soil samples were collected from 13 sampling sites in Lanzhou, Gansu Province, and Qiandongnan and Qiannan Prefecture, Guizhou Province. Among these, three species (*D. multiforme*, *D. firmibasis*, and *C. delicata*) occurred only in mixed forest, accounting for 27.27%; two species (*D. macrocephalum* and *H. tenuissimum*) occurred only in broadleaf forest, accounting for 18.18%; and one species (*D. giganteum*) occurred only in farmland, accounting for 9.09%. No species occurred in common in all five habitats (Fig. 5B).

The distribution of species with respect to relative abundance at different elevations and in different habitats is shown in the bidirectional bar chart (Fig. 5C). Three species were isolated from elevation 700–1,000 m, five species were isolated from elevation 1,000–1,300 m, six species were isolated from elevation 1,300–1,600 m, three species were isolated from elevation 1,600–1,900 m, and three species were isolated from elevation 1,900–2,200 m (Fig. 5C). Seven species were obtained in mixed forest, five species in coniferous forest, five species in broadleaf forest, three species in farmland, and one species in scrub (Fig. 5C). Two species of dictyostelids (*D. sphaerocephalum* and *D. mucoroides*) occur in common from four different forest types, accounting for 56.34% of the total number of species clones in this study.

#### Relationship between species of dictyostelids and environmental factors

Correlations of the dictyostelid species isolated from 13 sampling sites in Gansu and Guizhou Provinces with environmental factors were evaluated using RDA. Among the total environmental variance of the assemblage, 27.12% constrained was explained in RDA axis 1 (72.88%) and axis 2 (25.39%; Fig. 7). Temperature (*P* = 0.002), elevation (*P* = 0.002), and pH (*P* = 0.001) were the main impacting factors that determined the distributions of dictyostelid species in this study.

## DISCUSSION

### The significance of ecological studies of dictyostelids

One of the keys to dictyostelid studies, as opposed to other types of soil-dwelling single-celled eukaryotes, is the ease with which they can be cultured, identified, and enumerated. Ecological research of dictyostelids was first initiated by Cavender and Raper (25, 26). A central aim of ecology is the collection and interpretation of meaningful data and quantitative measurement of approximate biodiversity through conceptual models, mathematical models, computer simulations, and other methods in order to predict ecological processes, patterns, and dynamics, which is critically important for ecosystem health (32). The dictyostelids are understood to be permanent inhabitants of topsoil, where they represent a key component of the soil biota. To help assess the relationship between the occurrence of soil protozoans and soil ecosystem function (33), we should do a better job studying these factors that influence dictyostelid biodiversity. It is well known that different vegetation, ambient temperature, soil moisture, pH, available decomposing organic matter, and bacteria affect the dictyostelids species composition present in the soil at a particular locality (20).

### Distribution of diversity of dictyostelids at sampling points in Gansu and Guizhou

The present study was the first to collect soil samples from Gansu and Guizhou Provinces in 13 localities for isolation of dictyostelids in the laboratory. A total of 11 species of dictyostelids were isolated in this study, which were the first reports from the two provinces, with six species in Gansu and six species in Guizhou and one species in both provinces (Fig. 4 and 8). Almost all sampled areas revealed the presence of dictyostelids in this study, but only *D. firmibasis* and *D. multiforme* were regarded as uncommon, having been recorded previously from relatively few localities in China. *Cavenderia delicata* was the second report in China after that of Taiwan (Table 3).

**TABLE 3:** The current distribution in China of the species reported in this study

Species	Ecological environment	Distribution province	References	Species	Ecological environment	Distribution province	References
***D. purpureum***	*Acer ginnala* withered branches	Beijing	(34 – 37)	***H. tenuissimum***	Mixed forest soil	Henan	(37, 38)
	Lowlands	Taiwan			Broadleaf forest soil	Jilin	
	Mixed forest soil	Fujian		***H. pallidum***	Broadleaf forest soil	Taiwan	(35, 39)
	Pine forest soil	Henan			Deciduous forest soil	Jilin	(40, 41)
	Broadleaf forest soil				Lowlands		
***D. sphaerocephalum***	Coniferous forest soil	Heilongjiang	(37, 42)		Bamboo forest soil		
	Pine forest soil	Henan			Grassy topsoil		
	Broadleaf forest				Coniferous forest		
				***P. violaceum***	Broadleaf forest	Jilin	(34, 35, 37)
***D. mucoroides***	Deciduous forest soil	Beijing	(34, 43, 41)		Ginseng cultivation soil	Shanxi	(43, 39, 40)
	Decayed leaves	Jilin			Leaf rot	Taiwan	
	Coniferous forest soil				Lowlands	Henan	
	Tundra				Bamboo forest soil		
	Mixed broadleaf–conifer forest				Grassy topsoil		
***D. firmibasis***	Mixed forest soil	Jilin	(44)		Theropencedrymion soil		
***D. multiforme***	Animal dung	Qinghai	(28)		Coniferous forest		
***D. macrocephalum***	Lowlands	Taiwan	(35, 45)		Shrub soil		
	Topsoil of broad-leaved forests	Guizhou	(22)				
		Yunnan	(46)				
***D. giganteum***	Acacia tree and nursery	Taiwan	(36, 39)	***C. delicata***	Forest soil	Taiwan	(47)
	Broadleaf forest soil	Fujian					

The two species with the highest relative abundance, *D. sphaerocephalum* and *D. mucoroides,* comprised 56.34% of the total and are also widely distributed throughout the world. Among the previous research in Guizhou Province, only three dictyostelid species, identified as *Dictyostelium crassicaule*, *D. implicatum*, and *D. macrocephalum,* were listed by Yuan et al. (22), but there are no previous reports in the literature relating to the occurrence of dictyostelids in Gansu Province. In Guizhou Province of this study, with a smaller number of samples, the average density in the processed dictyostelid samples was 40 clones/g, compared with 44 clones/g for Gansu Province. The density of dictyostelids in Qinglong Mountain, Gansu Province, was the highest (122 clones/g) (Fig. 2), and the soil surface of Qinglong Mountain is covered with a thick layer of fallen leaves that prevent rapid changes in either temperature or moisture; therefore, this covering undoubtedly contributes to the diversity and the high frequency of dictyostelids (48).

The dissimilarity in the dictyostelid community structure between the two provinces was visualized using NMDS based on Bray–Curtis and PCA. The samples from the two provinces were clearly separated on the first two NMDS axes, and the communities were then clustered by localities within their respective provinces (stress = 0, ANOVA, *P* < 0.001, Fig. 6A). The PCA showed that the dictyostelid community structure was also clearly separated according to the provinces, and the contribution rate of two axes was 57.46% (Fig. 6B). The results suggest the significant differences in dictyostelid species between these two provinces.

### Influence of geographical factors on diversity of dictyostelids

#### Soil pH

The diversity of bactivorous dictyostelids has to be further explored with respect to environmental variables (49), specifically soil pH (50). For example, in forests of the French Alps, it was demonstrated that environmental factors (e.g., soil pH) other than the management of a forest are significantly correlated with the composition and richness of dictyostelids (51). The data reported in microbiological literature (52, 53) suggests that soil pH is one of the most important abiotic predictors in determining community composition. Also, in a recent paper (54), the authors provided valuable data on several edaphic factors such as soil pH, available nitrogen (AN), and available phosphorus (AP) and, on the basis of these data, concluded that pH was a key edaphic factor in determining bacterial community diversity and function. Our research shows the differences in the pH of the soil collected in the two provinces, where Guizhou is more acidic and the soil collected in Gansu province is more alkaline. The Venn diagram (Fig. 4) shows that the effect of soil pH on dictyostelid community composition is evident at levels of taxonomic resolution. The RDA results showed that environmental factors explained 98.27% of the variation in dictyostelid communities in soil samples from Gansu and Guizhou Provinces, with a cumulative contribution of 72.88% and 25.39%, respectively. This indicated that the soil pH (*P* = 0.001) had significant effects on the species diversity of dictyostelids (Fig. 7).

#### Elevation

In this study, 11 species of dictyostelids were isolated from soil samples collected at elevations of 700–2,200 m (Table 1; Fig. 8). The RDA results showed that elevation (*P* = 0.002) was the important predictor that explains the 77.6% constraint axis in the community composition (Fig. 7). Combining the Venn diagram (Fig. 5A) and the bidirectional bar chart (Fig. 5C), three species were isolated from elevation 700–1,000 m, five species were isolated from elevation 1,000–1,300 m, six species were isolated from elevation 1,300–1,600 m, three species were isolated from elevation 1,600–1,900 m, and three species were isolated from elevation 1,900–2,200 m. We found that the number of species of dictyostelids increased at first and then decreased with increasing elevation. This is consistent with Landolt et al. (55), who reported that elevation is negatively correlated with species abundance of dictyostelids. However, Paillet and Satre (51) found that the higher the elevation, the higher the dictyostelid species diversity. In the present study, there were six species of dictyostelids, with the largest number at elevations 1,300–1,600 m. These data suggest that middle-elevation sites were characterized by more species.

#### Temperature

The soil microbial community plays an important role in soil ecological function, and in the present study, temperature appeared to affect microbial activity and metabolic diversity of the dictyostelid communities. The temperature range when collecting in seven soil collection sites of Guizhou Province was 9°C–31°C, while the temperature range when collecting in six soil collection sites of Gansu Province was relatively lower, −6°C–13°C (Table 1). The RDA results showed that temperature (*P* = 0.002) was the important predictor that explains the 75.7% constraint axis; the environmental factor of temperature was significantly correlated with variations in dictyostelid communities (*P* < 0.05, Fig. 7). In addition, with respect to species composition, *D. mucuroides* and *D. sphaerocephalum* appeared in low-temperature habitats in Gansu (Fig. 5C), including Kushui Town (−6°C–13°C), Qinglong Mountain (−3°C–10°C), Jincheng Park, Baita Mountain, Lanshan Park, and Wuquan Mountain Park (−3°C–2°C), thus displaying that they have strong environmental adaptability and wide distribution. In previous research, Cavender (9) and Landolt et al. (56) recovered only *D. mucoroides* and *D. sphaerocephalum* from tundra (low-temperature soils); this suggested that the propagules had a strong resistance to unfavorable conditions and could survive in low-temperature environments, thus maintaining their stable properties.

### Influence of vegetation factors on the diversity of dictyostelids

When the distribution patterns of dictyostelids in different habitats were analyzed, seven species were recovered from mixed forests in this study, which suggests that species of dictyostelids appear most frequently in mixed forests (Fig. 5C). Certainly, among the described species, *D. mucuroides* and *D. sphaerocephalum* were distributed in four forest types of Gansu soil samples; these two species were the more frequent in Gansu soil samples. Other species such as *D. firmibasis*, *C. delicata*, *D. giganteum*, *D. multiforme*, *D. macrocephalum*, and *H. tenuissimum* were distributed in a single forest type, mostly mixed forest, and appeared to have a limited distribution among the localities sampled. *D. mucuroides* and *D. sphaerocephalum* are considered cosmopolitan species often encountered throughout the world (57, 58). This study also confirmed their characteristic as a widespread species in the world. Scientific studies have found that mixed forests are particularly interesting for resistance against the uncertainties of extreme climate development, as higher structural complexity and species interactions can be expected to be less affected in these forests compared to monospecific ones (59, 60). In a recent study, Zou et al. (41) studied the diversity of dictyostelids in Changbai Mountain of China and found that mixed broadleaf–conifer forests produced more isolates and species than broadleaf forests at the same elevation and also had the highest species richness. It is remarkable that *D. giganteum* and *D. sphaerocephalum* were reported again from farmland, consistent with previously reported species by Stephenson and Rajguru in agricultural soils in the United States (5). Although the data presented herein clearly document the occurrence of dictyostelids in Gansu and Guizhou soil, they also indicate the need for additional investigations of agricultural soil in China, which is still an understudied microhabitat.

### Cultivatability of soil microorganisms

The soil microbiota influences ecosystem function through the valuable contributions of organisms associated with any habitats (61), yet large amounts of microbial species remain undiscovered. It is estimated that as little as 0.3% of microbes can be cultivable, severely limiting their functional versus taxonomic diversity and thus communication among biologists (62). This includes soil eukaryotes. For example, dictyostelid fruiting bodies are microscopic and differentiated by their multicellular structures, which are difficult to observe directly except in laboratory culture. Therefore, the extent and geographic distribution of protist diversity remains largely unknown and a matter of controversial debate (63). Hagiwara (64) divided dictyostelids into “minute species” (1 mM), “small species” (2 mM), “medial species” (3.5 mM), “large species” (6 mM), and “gigantic species” (10 mM) according to their length. Most of the species isolated in this study are “large species” and grow robustly on the medium used to culture them. As a result, the frequency of some of these species such as *P. violaceum*, *D. sphaerocephalum*, and *D. mucoroides* recovered in this study likely may reflect not only the ease of cultivation in current laboratory cultures but also geographical ranges and macroecological pattern flexibility as well as probably high dispersal ability (18). It is therefore imperative that soil sampling in unknown regions should be increased in the future.

### Prospects for future research

Previous work has shown that complex seasonal dynamics occur in soil microorganisms (e.g., bacteria and fungi) (65, 66). In addition to this, the distribution and occurrence of dictyostelids is also affected by seasonal fluctuations. In dry mesic forests, the population of dictyostelids in spring and autumn has been shown to be about double that in summer and winter (2). The soil of Gansu Province in this paper was collected from winter; the soil of Guizhou Province was collected from autumn and winter; however, the isolated dictyostelids were not very different. However, the region may not have been sampled enough. Certainly, the soil habitat is a complex system influenced by various environmental factors that contain a vast number of microorganisms and harbor a substantial fraction of the planet’s biological diversity (67, 68). Species of soil protists exhibit very different global-scale biogeographical patterns (69). High-throughput sequencing methods are now instrumental in decoding protist community diversity and revealing new microbial lineages (70, 71). Overall, future research should combine culture and morphological-based approaches with genomic methods to characterize patterns of amoeboid protist community diversity and contributions to ecosystem functioning in single soil types, spatial structure, and sampling points, which likely improve our understanding of their ecological importance in soils.

## Data Availability

The SSU rRNA gene sequences of the dictyostelid isolates in this study are available in GenBank under accession numbers OP311638, OP310835, OP310944, OP314172, OP311412, OP390235, OP390237, OP390269, OP390285, OP390455, OP390454, OP390643, OP390737, OP392881, OP392892, OP392943, OP310822, OP392986, OP392974, OP392991, OP392994, OP311593, OP310802, OP310985, OQ348111, OP310969, OP310943, OP311669, OQ345561, OP310821, OP311727, and OP310071.
